# Supplementary material for: Evaluating the Feasibility and Reliability of Remotely Delivering and Scoring the North Star Ambulatory Assessment in Ambulant Patients with Duchenne Muscular Dystrophy
Source: Children (Basel). 2022 May 16;9(5):728. doi: 10.3390/children9050728 (PMC9139300; doi:10.3390/children9050728)
Supplement: Supplementary file 1 [file children-09-00728-s001.zip › children-1670399-supplementary.pdf]

Supplementary S1

Consent:

Title of study: **Evaluating the feasibility of remotely delivering and scoring the North Star Ambulatory Assessment (NSAA) in ambulant patients with Duchenne Muscular Dystrophy (DMD)**

Site: Robert Jones and Agnes Hunt Foundation Trust Hospital

Introduction:

The purpose of this study is to determine whether the NSAA can be performed and accurately scored remotely by means of video link. This information sheet will detail how we propose to do so.

Despite the country having been in lockdown since 23rd March 2020 due to the SARS-COV-2 pandemic there is still a clinical need to regularly monitor patients with DMD. This enables us to track progression or decline in functional activities, monitor effectiveness of corticosteroid regimes and alter dosage as required. Standards of care recommend that boys with DMD be assessed every six months.

You will already be familiar with the NSAA as we have scored your son at least twice in our clinical setting. It is a validated and reliable method used for assessing the functional abilities of boys with DMD. The NSAA is a 17 item rating scale specifically developed by the North Star Clinical Network for Paediatric Neuromuscular Disease to measure function in ambulant children with DMD.

How we propose to carry out the study:

Your son will be one of ten boys with a confirmed diagnosis of DMD asked to take part in the study. Their previous two NSAA scores from clinic appointments will be taken as a base line reference.

If, as a family, you are happy to go ahead with the study we will send you details of the NSAA tasks that will be carried out at your home. This will include advice on appropriate dress and equipment requirements, including suitable step and chair height, and a 10m walk/run area. For any item that you are unable to provide the equipment for we will score 0, however the previous scores will also be altered accordingly to reflect this.

The video link will be set up via a secure online platform called Attend Anywhere (AA). You will be sent additional information regarding the setup of this user friendly software.

You will already be familiar with our Physiotherapy team carrying out the assessment, and we will guide you through in a similar way to that if you were in clinic with us. The way in which the items are performed and the tasks scored will be in keeping with the assessments ordinarily carried out in a clinic environment. The score will then be compared to the two previous scores and a percentage change will be calculated. The Physiotherapist carrying out the NSAA via AA will not know the previous two scores.

Does my son have to take part?

We would like to invite you to allow your child to take part in this study, but it is up to you to decide whether you want your son to take part. However, if it is your son's choice to not take part then he will not be included in the study even with your consent. You may change your mind at any time during the study. Your son may leave the study at any time.

If you decide to join, you must sign at the end of this form to show that you agree for your son to take part in the study. This is called "giving consent". The study might be stopped at any time. If this happens we will give you the reason at that time.

If you and your son decide not to take part in this study there is no penalty or change to the standard of care that your son receives. You will be given a copy of this information sheet and your signed informed consent to keep. Your son may also sign a similar and more simplified form.

What will happen to my son if he takes part?

As outlined above, the NSAA will be carried out at home via video link. This will be the same version of the assessment that he would already have carried out at least twice during clinic appointments at the hospital. You will be sent all necessary information to help you perform the assessment prior to the video call.

ow long will it take?

We estimate the assessment to take no longer than twenty to thirty minutes. In some cases this may be a little longer, depending on possible technology issues and equipment available

Are there any risks involved with taking part?

As when in clinic, your son may need assistance to keep him safe during some of the tasks. This will be encouraged to reduce the risk of falls and injury; we can advise further during each task.

Will information and personal information from this study be kept confidential?

All the data/scores collected will be held securely and in confidence. The scores obtained from the NSAA will be entered in to the North Star database, as usual, for which you have already provided consent. The boys participating in the study will not be identifiable. The data collected on NSAA scores may be disseminated to doctors and physiotherapists, be published in medical journals and/or shown on posters at medical conferences.

Further information and contact details:

We hope this information is sufficient to enable you to decide whether you want your son to participate in the study. Please ask any questions you may have about this study before signing the consent form. If you have any questions or medical problems during the study, please contact:

Professor Tracey Willis; 01691 404047

Neuromuscular Clinical Specialist Physiotherapist Nick Emery; Contact 01691 404493

You can also make a formal complaint through the hospital procedure or contact someone who is independent of the research study.

Consent:

Please initial each box alongside the following statements and sign at the bottom

☐

I have read and understood the information regarding this study. { }

I have had the opportunity to discuss the study and ask questions in a language that I understand. I am satisfied with the answers. { }

I have had time to make my decision. { }

I understand that my son's participation is voluntary and that my son and I are free to withdraw at any time without giving any reason, and that my son's medical care and legal rights will not be affected. { }

I agree with collection, processing, reporting and transfer NSAA data as required by the study. { }

I agree to our family doctor and other treating specialists being informed of my son's participation in the study. { }

I agree for publication and dissemination of the information at meetings with other doctors to help inform others on the usefulness of this facility (your child will not be identifiable). { }

I agree that my son takes part in the above study. { }

Parents/Guardian Name:

Print…………………………Sign………………………………Date…………
…

Professor Tracey Willis:

Print…………………………Sign………………………………Date…………
…

Nicholas Emery (physiotherapist):

Print…………………………Sign………………………………Date…………
…

Supplementary S2.

NSAA Brief Test Detail

Activity	2	1	0	Score
1. Stand	Stands upright, still and symmetrically, without compensation (with heels flat and legs in neutral) for minimum count of 3 seconds	Stands still but with some degree of compensation (e.g. on toes or with legs abducted or with bottom stuck out) for minimum count of 3 seconds	Cannot stand still or independently, needs support (even minimal)	
2. Walk	Walks with heel-toe or flat-footed gait pattern	Persistent or habitual toe walker, unable to heel-toe consistently	Loss of independent ambulation – may use KAFOs or walk short distances with assistance	
3. Stand up from chair	Keeping arms folded. Starting position 90º hips and knees, feet on floor/supported on a box step.	With help from thighs / push on chair / prone turn or alters starting position by widening base.	Unable	
4. Stand on one leg – right	Able to stand upright in a relaxed manner (no fixation) for count of 3 seconds	Stands but either momentarily or with trunk side-flexion or needs fixation e.g. by thighs adducted	Unable	
5. Stand on one leg - left	Able to stand upright in a relaxed manner (no fixation) for count of 3 seconds	Stands but either momentarily or with trunk side-flexion or needs fixation e.g. by thighs adducted	Unable	
6. Climb box step - right	Faces step – no support needed	Goes up sideways / rotates trunk / circumducts hip or needs hand for balance	Unable	
7. Descend box step – right	Faces forward, steps down controlling weight-bearing leg. No support needed	Sideways, skips down or needs hand for balance	Unable	
8. Climb box step - left	Faces step – no support needed	Goes up sideways / rotates trunk / circumducts hip or needs hand for balance	Unable	
9. Descend box step - left	Faces forward, steps down controlling weight-bearing leg. No support needed	Sideways, skips down or needs hand for balance	Unable	
10. Lifts	In supine, head must be lifted in mid-line. Chin	Head is lifted but through side flexion or with no neck	Unable	

Updated Physiotherapy Manual Version 2.0 May 2017

28

Activity	2	1	0	Score
head	moves towards chest	flexion (protracts)		
11. Gets to sitting	Starts in supine – may use one hand / arm to push up	Uses two arms / pulls on legs or turns towards floor.	Unable	
12. Rise from floor	No evidence of Gower's maneuver.	Exhibits at least one of the Gower's components described– in particular rolls towards floor, and/or use hand(s) on legs	(a) NEEDS external support of object e.g. chair OR (b) Unable	Timed
13. Stands on heels	Both feet at the same time, clearly standing on heels only (acceptable to move a few steps to keep balance) for count of 3	Raises forefoot on both feet – all metatarsal heads off ground – or clearly dorsiflexes one foot only	Unable	
14. Jump	Both feet at the same time, clear the ground simultaneously	One foot after the other (skip) or does not fully clear both feet at the same time.	Unable	
15. Hop right leg	Clears forefoot and heel off floor	Able to bend knee and raise heel, no floor clearance	Unable	
16. Hop left leg	Clears forefoot and heel off floor	Able to bend knee and raise heel, no floor clearance	Unable	
17. Run (10 m)	Both feet off the ground (no double stance phase during running)	'Duchenne jog' or fast walk	Walk	Timed
TOTAL=	/34			

Updated Physiotherapy Manual Version 2.0 May 2017

29

Supplementary S3.

The video link will be via a secure site approved by the National Health Service England and only the Doctors and physiotherapist will be able to see your son and the score collected. The test should take no longer than 30 minutes.

A guide to what you will need for the video NSAA:

1. A laptop computer with camera, camera phone, iPad or similar device and a well-lit room

2. Connection to the internet via WiFi

3. Your son bare foot and in shorts

4. A chair so that when sat down your sons knees and hips are at right angles and his feet are flat on the floor (if you don't have this don't worry)

5. A step that is 15cm (6 inches in height) (if you don't have this don't worry)

6. A clear space (no furniture within arm's reach)

7. A flat area that is 10 metres long (33 feet) (don't worry if this is not possible). Or a flat area that your son can walk/run down. See enclosed template for making a cone.

The following is a guidance sheet help to you position your son for the assessment.

Test item	Start position	Instructions
Stand	Standing with feet facing forward and one hands width apart	Can you stand tall
Walk	Walk no shoes for 10 steps	Walk how you normally walk
Stand up from chair	Sitting with hips and knees at right angle	Stand up from the chair. Try not to push on your legs or the chair
Stand on Right leg	Try and balance on one leg not holding on to anything	Can you stand on your right leg for as long as you can
Stand on Left leg	Try and balance on one leg not holding on to anything	Can you stand on your left leg for as long as you can
Step up on box Right leg	Stand facing box	Can you step up on to the boxing you Right leg
Step down from box Right leg	Stand on top of box facing forward	Can you step down from the box using your Right leg
Step up on box Left leg	Stand facing box	Can you step up on to the boxing you Left leg
Step down from box Left leg	Stand on top of box facing forward	Can you step down from the box using your Left leg
Get to sitting	Lying on back on floor, arms by side, legs together	Can you get from lying down to sitting
Rise from floor	Lying on back on floor, arms by side, legs together	Can you get up from the floor as fast as you can
Lifts head	Lying on back on floor, arms across chest, legs together	Can you lift your head and look at your toes
Stands on heels	Standing on floor	Can you stand on your heels, lift the front of your feet
Jump	Standing on floor feet fairly close together	How high can you jump
Hop on Right leg	Standing on floor on Right leg (if able)	Can you hop on your Right leg
Hop on Left leg	Standing on floor on Left leg (if able)	Can you hop on your Left leg
Run (10 metres)	Starting with toes in line with the cone.	Run as fast as you can to the other cone

Supplementary S4

Certificate of institutional approval attached separately.

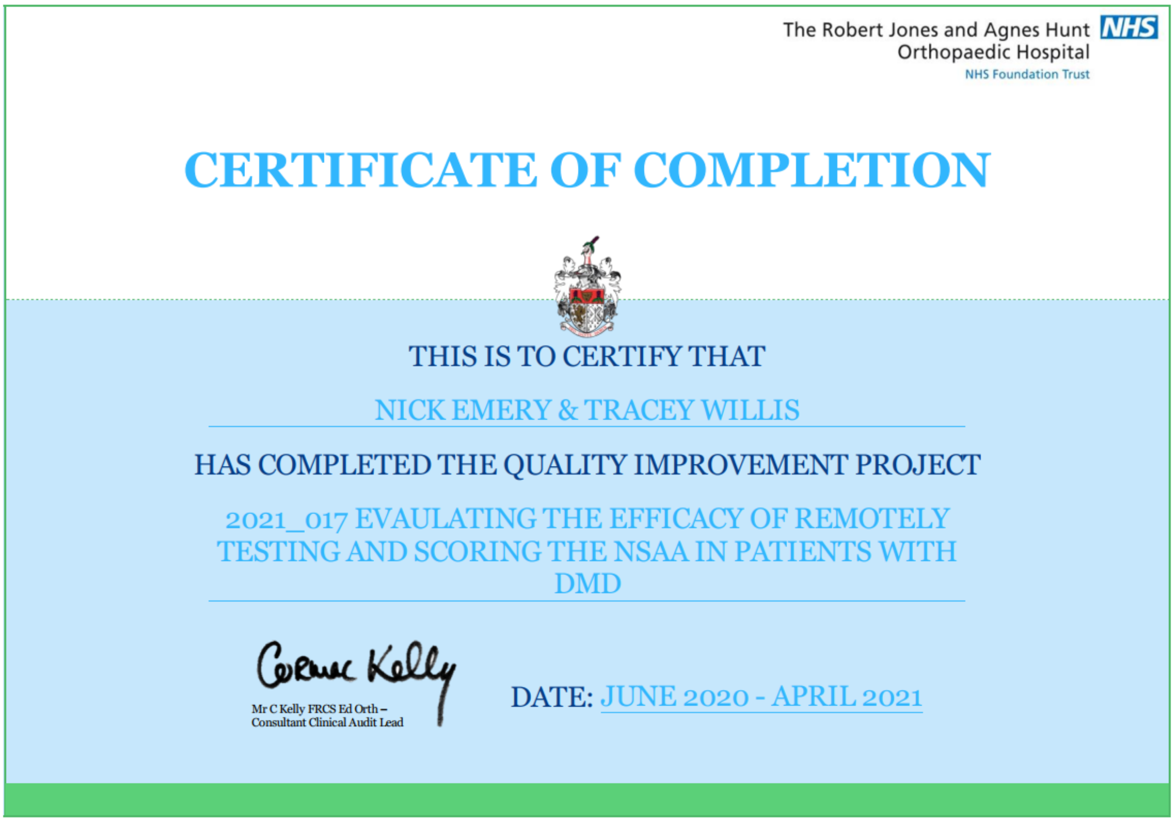

The Robert Jones and Agnes Hunt Orthopaedic Hospital
NHS Foundation Trust
NHS

CERTIFICATE OF COMPLETION

THIS IS TO CERTIFY THAT

NICK EMERY & TRACEY WILLIS

HAS COMPLETED THE QUALITY IMPROVEMENT PROJECT

2021_017 EVAULATING THE EFFICACY OF REMOTELY TESTING AND SCORING THE NSAA IN PATIENTS WITH DMD

Cormac Kelly

Mr C Kelly FRCS Ed Orth – Consultant Clinical Audit Lead

DATE: JUNE 2020 - APRIL 2021
